# Leadership in intellectual disability practice: design, development, and evaluation of a programme to support practice

**DOI:** 10.1186/s12913-024-11124-7

**Published:** 2024-05-28

**Authors:** Owen Doody, Maeve O’Halloran, Eileen Carey, Marie Kilduff, Ann Gilmartin, Ruth Ryan

**Affiliations:** 1 Health Research Institute, Department of Nursing and Midwifery, University of Limerick, Limerick, Ireland; 2 Intellectual Disability Services, Clare, Ireland; 3 National Clinical Leadership Centre for Nursing and Midwifery, Health Service Executive, Dublin, Ireland; 4 National Clinical Leadership Centre for Nursing and Midwifery, Health Service Executive, Sligo, Ireland

**Keywords:** Intellectual disability, Practice leadership, Nursing, Social care, Leadership

## Abstract

**Background:**

Intellectual disability services have and continue to experience changes in service provision. This has an implication for leadership in practice as the quality of leadership has a direct influence on staff practice and care provided.

**Aim:**

To design, deliver, and evaluate a leadership programme for nurse and social care managers in Ireland.

**Design:**

An accredited programme was designed based on evidence from literature, practice, and national expertise. A cross-sectional survey was used to collect information on the attitudes and behaviour of participants before commencing and after completing the programme. Data from the questionnaires were analysed using SPSS and open-ended questions were analysed using content analysis.

**Setting:**

Intellectual disability services.

**Participants:**

102 participants completed the programme and survey.

**Methods:**

Pre-post survey and reported using the CROSS guidelines.

**Results:**

Participants’ expectations were rated highly, and all items scored higher in the post-survey. Qualitative data was overall positive regarding opportunities for more time to work through each aspect of the programme. The key learning was through the forum day where participants shared their group projects.

**Conclusions:**

Overall, the programme was positively evaluated and through engaging with the programme participants’ perceptions moved from seeing leadership as mostly task-oriented to realising that qualities such as good communication, person-centredness, advocacy, supporting, role modelling, and empowering are key to leadership.

**Supplementary Information:**

The online version contains supplementary material available at 10.1186/s12913-024-11124-7.

## Introduction

Irish intellectual disability services have experienced a changing landscape of service provision over the past three decades [1]. This evolving landscape has seen a movement towards community settings, a changing culture, the provision of personalised supports, and a changing demographic profile [2]. Irish intellectual disability services are primarily funded through a combination of government allocations, health service budgets, and contributions from non-profit organisations. In Ireland, the context of practice leadership in intellectual disability services is shaped by various factors, including historical, societal, and political influences. The provision of support for people with intellectual disability has undergone significant changes over the years, often in response to various pressures and challenges within the healthcare system and broader society. One significant pressure for changing support provision has been the occurrence of hospital scandals, which have highlighted shortcomings in the care and treatment of individuals with intellectual disability. These scandals, exposed instances of neglect, abuse, and substandard living conditions within residential care settings and highlighted the urgent need for reform and improvement in the delivery of services for individuals with intellectual disability. Key policy documents and strategies guiding change in Ireland include; Time to Move on from Congregated Settings [3], Progressing Disability Services for Children and Young People [4], New Directions [5], A National Framework for Person-Centred Planning in Services for Persons with a Disability^6,^ UNCRPD [7], Assisted Decision Making (Capacity) Act [8] and Sláintecare [9]. These policy documents and strategies have implications for intellectual disability services in terms of leadership, professional care, and support skills required to address the needs of people with intellectual disability (and their families). Key principles within these policies/strategies are person-centred care, rights, quality, safety, and risk management and in line with Irish policies, all designated centres for people with disabilities (children and adults) must be registered with the Health Information and Quality Authority (HIQA) since November 2013.

All registered designated centres are subject to inspections by HIQA inspectors who examine and evaluate services to safeguard that they comply with the National Standards for Residential Services for Children and Adults with Disabilities [10]. The legislation underpinning the standards also necessitates that a Person-in-Charge (PIC) for each designated centre be appointed and that the PIC should have relevant clinical and leadership skills and experience to safeguard the effective administration of their service and deliver safe, effective care to support clients/service users and their families. Under the most recent HIQA guidance [11], a PIC should have the appropriate qualifications to fulfil the post and have the relevant skills and experience to effectively manage the size of the centre, the number of residents, and the assessed needs of the residents. A PIC may be over more than one designated centre and variations exist in the number of designated centres and the number of residents. However, the specific requirement for leadership knowledge is vague with a requirement of 3 years in a management or supervisory role.

In the context of practice leadership for frontline managers, such as PICs in intellectual disability services in Ireland frontline managers often lead interdisciplinary teams comprising various professionals, including nurses, social care workers, psychologists, therapists, and support staff. Nurses in intellectual disability services may indeed work in both social care settings, such as community residences or day centres, and residential care settings, depending on the needs of the individuals they support. Ireland’s intellectual disability services encompass a mixed economy of care, involving voluntary, private, and state-managed organisations, and comprise a diverse range of supports tailored to meet the needs of individuals across the lifespan. Services vary in size, with some supporting a small number of residents in shared living arrangements, while others may accommodate larger groups. The exact number and size of services fluctuate across regions based on population demographics, local demand, and available resources.

The significance of leadership has been emphasised in the literature [12, 13] and National reports [14, 15]. Evidence within the international literature indicates that the quality of front-line management and leadership is a multi-faceted and complex role and has a direct influence on staff practice [16, 17]. In recognition of the impact of leadership and the rapidly changing health and social care environments, there is a need to support managers/leaders working in intellectual disability services to provide support in the form of education and training to meet the PIC requirements/role. The National Clinical Leadership Centre for Nursing and Midwifery (NCLC) commissioned the authors to design, deliver, and evaluate a leadership programme for Clinical Nurse Manager (CNM) and Social Care Leader (SCL) grades with PIC responsibilities. The programme was developed based on preliminary work with CNMs and SCLs from intellectual disability services to identify core elements and priority areas of leadership competencies in intellectual disability health and social care leadership roles. This co-design element informed the development of the NCLC’s seven competencies (self-awareness, empowerment, advocacy, communication, decision-making, quality and safety, and teamwork) that underpinned the programme. To support the co-design of the programme an experienced intellectual disability nurse with experience in inspections, leadership, management, and consultancy work supported the design, delivery, and evaluation process (MO’H).

The need for leadership within all staff in the health and social care system has been expressed [18] and within intellectual disability, staff are from either a nursing or social care context who respond to situations that require intervention. Such responses require initiative, resourcefulness, motivation, an ability to solve problems, active awareness, persistence to achieve goals, and communication with team members i.e., leadership [19]. Effective leadership improves client/service user satisfaction and care outcomes [20, 21] and while leadership is customarily considered in the context of upper management roles [22], leadership and teamwork within practice has gained interest [23]. The move away from a transactional model of leadership which emphasised a hierarchical top-down management structure^24,^ has seen a shift to an approach that seeks to develop; the ethos, values, and motivation of team members, and effective relationships, communication, empowerment, and engagement of all staff [25]. Also, it is recognised that health and social care organisations, should support leadership that focuses on team building, work relationships, promoting participation, and the co-creation and facilitation of care processes [19]. 

Addressing leadership and supporting leaders is fundamental to intellectual disability organisations/service providers as they face numerous challenges that impact the effectiveness, efficiency, and sustainability of service design and delivery. Such impacts are seen in licensing requirements, accreditation standards, performance monitoring, and consumer expectations and this calls for a new kind of leadership away from heroic individual leaders to a model of distributed leadership across organisations and systems [26]. As we face the future, we must continually question if standard practices are working well, and how we will enhance personal outcomes and generate organisation outputs that reflect a good return on investment. Given that effective leadership is vital to prevent poor standards and ensure people with intellectual disability experience significant improvements in their lives there is a need for new leadership approaches and thinking within health and social services [27]. This paper describes the design, delivery, and evaluation of a leadership programme for CNMs and SCLs with PIC responsibilities in Ireland.

## Methodology

### Programme design and delivery

Within the programme design stage, the academic leads, members of the NCLC, and the CNMs and SCLs involved in the preliminary work assisted in co-designed the programme based on the evidence from the literature and the NCLC seven leadership competencies. Within the co-design process, it was agreed academic and professional accreditation was important to value participant engagement and work. To acknowledge this firstly, the programme was submitted to the Nursing and Midwifery Board of Ireland (NMBI) to gain professional accreditation (5.5 days) and the maximum 35 Continuing Education Units (CEUs). The second accreditation process involved presenting the programme in a module format (9 Credit (ECTS) Level 9) for academic accreditation which would enable access to postgraduate level 9 courses for those who do not hold a degree level qualification (programme learning outline/outcomes Table 1).

**Table 1:** Programme learning outcomes and outline

**Programme learning outcomes** • Develop knowledge and skills in quality improvement using a quality improvement model. • Appraise the difference between ‘management’ and ‘leadership’ and outline different management/leadership models and styles in developing and demonstrating the responsibility, authority and accountability associated with the role of PIC. • Describe the principles of change management/leadership and strategic planning. • Discuss the influences of power dynamics within an organisation. • Design a plan for implementing change within a practice setting. • Demonstrate awareness of the continuous nature of self-assessment. • Reflect on experiences and investigations. • Appreciate key influences in managerial and leadership styles and understand the importance of resilience for personal and professional well-being. • Establish a team and demonstrate team building skills and networking abilities. • Expand leadership skills in quality and safety in services. • Foster and embed a culture of empathy in delivering person centred care and support. • Develop clinical leadership competencies and actively engage with the clinical leadership competencies. • Develop and demonstrate team building skills and networking ability. • Demonstrate leadership in quality and patient safety improvements in services. • Understand the importance of resilience for personal and professional well-being. • Undertake a work-based initiative using quality improvement methodologies.
**Programme outline** • Introductions and welcome, • Programme Registration, • Programme content and outline (including resources), • Needs led sessions (roles, responsibilities, clarification), • Self-awareness (values, beliefs, strengths, weaknesses, development), • Policies guiding and supporting leaders, • Responding to change in disability services: the role of leadership, • Differentiate between ‘management’ and ‘leadership’ – thinking as you go forward, • Engagement and empowerment of clients/service users, • Enabling a culture of person-centredness, • Person-Centred Planning (PCP) and empowerment in practice-evidence of successes and not so successful implementation, • Guidance and discussion on the design of staff engagement interventions as part of your work to improve quality improvement, • Rights approaches in health and social care, • Supporting decision making and assessment of capacity: Irish Legislation, • Assisted Decision Making (Capacity) Act 2015 as it relates to Intellectual Disability, • Implementing the ADM(C) Act - challenges and perspectives, • Embedding the ideology of the UNCRPD in disability services: discussion forum, • Advocacy, advocating for self, clients/service users, staff, and role of advocacy in intellectual disability services, • Teamwork and team approaches, • Critical reflection for change, • Preparing for HIQA inspections, • Future roles, • Roles and function of leadership and accountability for good health and social care, • Clinical decision making, • Quality care in intellectual disability services, • Quality improvement plans as part and parcel of practice, • The role of the person in charge, • Guidance and discussion on quality improvement and leadership competencies, • Forum day – presentations to groups, services, interested parties.

The programme philosophy aimed to enhance participants’ confidence and skills to enable them to effectively apply these skills in developing plans for improvement or change. Moreover, to empower them to initiate initiatives in practice that prioritise the delivery of person-centred care and support. Participants were encouraged to draw on their experiences of practice and explore theories and models of leadership and management within the context of health and social care practice. Participants explored aspects of PIC leadership and management, focusing on learning, quality, and improvement e.g., communication, empowerment, decision-making, operational management, effective governance, and administration of the designated centres, health promotion, and protection activities, and supporting a safe quality service.

Participants were recruited through national intellectual disability services, where a poster advert for the programme was sent for distribution within the organisation. To avail of a place participants had to have service support and release to attend the 5.5-day programme and other than travel the programme was free to the service and participant. The programme delivery was coordinated by the lead author (OD) and facilitated in person by the academic leads, guests with leadership roles in health and social care services or leadership centres, and the intellectual disability nurse as part of the co-design team. Participants would reflect on their learning and document their professional knowledge through reflective inquiry. The accumulation of learning would be presented as group projects in a half-day event (forum day) open to service providers and staff. The forum day consisted of an opening presentation to mark the focus of the programme, a speaker from the NCLC, the group presentations, an invited inspirational guest speaker on leadership, and a closing event. Projects for the forum day (Table 2) were decided upon based on identifying areas of practice and leadership that participants wished to address and develop within their practice and translated across services so the group could benefit and participate. This process ensured initiatives spanned across services and was supported and mentored by the intellectual disability nurse on the co-design team.

**Table 2:** Projects undertaken

**COHORT 1**
• Providing support and guidance to staff to ensure person centred practices are upheld for those that we support. • Putting the HIQA into Team Meeting. • The Right to choose where to live. • Implementing a supervision process, within day and residential services in conjunction with the staff team. • Reviewing the time spent on paperwork in the evening; being with the people we support. • Development of a user-friendly template for goal setting for the people we support. • Supporting debriefing to promote a culture of support and learning: development of an incident support and learning review template. • Assessing and supporting staffs’ readiness for HIQA. • Someone at the door: Quality improvement tool to support client through the HIQA inspection process. • Quality improvement for restrictive procedures. • Improving weekly handover and reminders. • Developing a communication checklist for handovers. • Promoting staff awareness of personal development plans. • Individual transitions plan, utilizing technology to support a service user approach. • Making a good impression: a person-centred approach to introduce new staff within a service. • The implementation of a daily handover sheet. • Quality of life outcomes for transition. • Making supervision work for you. • Assisted Decision Making Tool: A template for staff to build capacity of people with intellectual disabilities to make their own decisions. • Introduction of Clinical peer supervision in a Social Care setting. • Managing Concerns of frontline staff regarding Inspection processes. • Staff stress: Are staff stressed. • Improving the staff team meeting process. • Improving one-to-one clinical supervision practices.

### Evaluation process

The forum day was the evaluation process for the project undertaken and this was assessed by academic colleagues, members of the NCLC, and service managers based on the value of the project to practice, its transferability across service, and its impact on client/service users lives. Permission to conduct the study was granted by the lead author’s University Research Ethics Committee (Education and Health Sciences, Research Ethics Committee), and participant’s rights to confidentiality and anonymity were upheld throughout the conduct of this study. Participants were Clinical Nurse Managers (1 or 2) or Social Care Managers with PIC responsibilities working in residential services for people with intellectual disability in Ireland. Four programmes were delivered over two years February to June and September to January with 25 to 30 participants per cohort. In total across the four programmes 110 participants registered of which 102 completed the programme. Attrition of the eight participants was due to personal reasons (*n* = 1), issues regarding release from work due to staff shortages (*n* = 6) and moving to a new job (*n* = 1). A cross-sectional survey tool developed by the researchers for this study (Supplementary file S1) was used to collect information on the attitudes and behaviour of the participants before commencing and after completing the programme. The survey tool was developed for this study as this was a new programme and creating the survey tool allowed for the tailoring of questions to address the specific evaluation and unique elements of the programme. A hard copy format was distributed before the programme commenced and after the forum day and returned by participants on the day or by post. Data collection and analysis were managed by the intellectual disability nurse who was part of the co-design team. The questionnaire consisted of 53 questions divided into five sections. Section one covered demographic details (Q1-Q13), section two addressed aspects before commencing the programme (Q14-Q22), section three addressed expectations during the programme (Q23-Q33), section four addressed expectations upon completion of the programme (Q34-Q52) and section five (Q53) were an open text for additional comments to add depth and give meaning to participants experience. Data from the questionnaire pre/post were analysed using SPSS where descriptive statistics and a Cronbach value (α) were calculated to check the reliability of each subsection. Elo and Kyngäs [28] systematic approach to content analysis was used for categorising and analysing the textual data to identify patterns, themes, or relationships.

## Results

As part of the programme evaluation, a pre-post self-report survey was used to capture demographic details, perceptions regarding commencing the programme, expectations during the programme, and expectations upon completion of the programme. Of the participants on the programme, 15 (14.7%) were male and 87 (85.3%) were female with 93 (91.2%) working full-time and the remaining 9 (8.8%) working part-time. From a working pattern perspective, 52 (51%) worked day duty with no weekends, 31 (30.4%) worked day duty with weekends, and 19 (18.6%) worked shift work including nights. General demographic details of age, role, qualification, and years of experience are presented in Table 3. Cronbach’s alpha was calculated to measure the internal consistency of scale reliability with the three scales performing well for the pre and post survey; scale 1 perceptions regarding commencing the programme, α (0.689 pre, 0.860 post), scale 2 expectation during the programme α (0.712 pre, 0.787 post) and scale 3 expectation upon completion of the programme α (0.960 pre, 0.943 post).

**Table 3:** Participants’ demographic detail (*n* = 102)

Age	20–30 years *n* = 16 (15.7%) 31–40 years *n* = 33 (32.4%) 41–50 years *n* = 41 (40.2%) 51–60 years *n* = 11 (10.8%) 60 + years *n* = 1 (1%)	Years’ experience in intellectual disability care	1 to 5 years *n* = 5 (4.9%) 6 to 10 years *n* = 26 (25.5%) 11 to 20 years *n* = 40 (39.2%) 21 + years *n* = 31 (30.4%)
Qualification	Certificate *n* = 13 (12.7%) Degree *n* = 62 (60.8%) Postgraduate Diploma *n* = 19 (18.6%) Masters *n* = 8 (7.8%)	Role	Registered Nurse *n* = 9 (8.8%) Nurse Manager *n* = 44 (43.1%) Clinical Nurse Specialist *n* = 2 (2%) Social Care Worker *n* = 3 (3.9%) Social Care Manager *n* = 44(43.1%)

Scale one measured participants’ perception regarding the programme, and pre- and post-program delivery. Participants’ expectations were rated highly and regardless of the high expectation evident in the pre-survey all items did perform and scored higher in the post-survey. Of note was that participants rated the question on the programme assisting in the prospect of promotion lowest (58.8%) in the pre-survey and while it remained lowest in the post-survey it had increased to 91.2% indicating the potential and perceived value of the programme for the participant’s career advancement. The overall scoring of each item on the scale is presented in Table 4.

**Table 4:** Survey results

	Pre Level of agreement, Mean, SD	Post Level of agreement, Mean, SD
**Perceptions regarding the programme**		
**Commencing the programme**		
Programme information accessible	81.4%, 1.99, .764	99.0%, 1.47, .521
Sufficient information to decide to enrol	84.3%, 1.95, .750	95.1%, 1.57, .668
Programme will assist me in my practice	97.1%, 1.35, .624	100, 1.54, .501
Programme will assist me in my future career	97.1%, 1.31, .526	98%, 1.55, .538
Programme, improve my knowledge and skill	96.1%, 1.40, .601	100%, 1.36, .483
Programme, assist my prospect of promotion	58.8%, 2.3, 1.032	91.2%, 1.45, .654
Programme part of my personal development	94.1%, 1.49, .609	98%, 1.37, .525
Programme, give me greater job satisfaction	88.2%, 1.75, .681	96.1%, 1.54, .608
Programme, benefit my organisation	91.2, 1.63, .674	94.1%, 1.46, .640
**Expectation during the programme**		
**During the programme**		
Content appropriate to my practice	97.1%, 1.57, .554	98.0%, 1.53, .540
Introduction to all relevant resources	95.1, 1.64, .577	98.0%, 1.60, .567
Resources available	96.1%, 1.64, .594	99%, 1.49, .522
Difficulty to adjust to academic life	39.2%, 2.89, 1.168	78.4%, 1.81, 1.115
Apply learning to practice	95.1%, 1.59, .650	97.1%, 1.59, .551
Moodle will be a valuable resource	87.3%, 1.69, .844	91.1%, 1.70, .729
Facilitators supportive	97.1%, 1.58, .553	99.0%, 1.27, .470
Asking questions related to topics	96.1%, 1.58, .571	100%, 1.39, .491
Manage time well	88.2%, 1.79, .635	90.2%, 1.73, .692
Assessments appropriate	91.2%, 1.78, .591	96.1%, 1.59, .635
Aware of the time necessary	84.3%, 1.83, .705	93.1%, 1.62, .704
**Expectation upon completion of the programme**		
**Completion of the programme**		
Gain knowledge relevant to person in charge	94.1%, 1.61, .600	95.1%, 1.40, .679
Analytical skills will develop	94.1%, 1.72, .603	97.1%, 1.58, .588
Gain a comprehensive view of client/service user care	88.2%, 1.77, .673	93.1%, 1.52, .728
Aware of the evidence behind practice	92.2%, 1.65, .624	95.1%, 1.49, .656
Develop my skills in evaluating evidence	88.2%, 1.74, .688	93.1%, 1.45, .684
Become skilled in evaluating my own practice	91.2%, 1.68, .662	97.1%, 1.52, .593
Able to implement change in my practice area	91.2%, 1.71, .623	98%, 1.61, .539
Will positively impact on client/service user care	94.1%, 1.55, .607	97.1%, 1.52, .593
Confident talking about care	85.3%, 1.73, .706	91.2%, 1.48, .741
Apply my learning in my workplace	97.1%, 1.47, .558	100%, 1.40, .493
Confidence communicating to colleagues	93.1%, 1.58, .620	99.0%, 1.48, .521
Confidence in speaking at team meetings	86.3%, 1.71, .698	92.2%, 1.58, .724
Share my knowledge with other colleagues	92.2%, 1.58, .636	99.0%, 1.44, .555
Become more innovative in my practice	89.2%, 1.71, .654	92.2%, 1.45, .753
Confident advocating for clients/service users	89.2%, 1.68, .692	93.1%, 1.42, .710
Programme will positively impact my work	94.1%, 1.56, .606	100%, 1.49, .502
Programme increase work satisfaction	88.2%, 1.73, .692	92.2%, 1.51, .700
Programme positively impact on my career	85.3%, 1.76, .747	95.1%, 1.55, .591
Programme increased my responsibility	53.9%, 2.31, .975	87.3%, 1.61, .810

The second scale measured participants expectations regarding the programme, pre- and post-programme delivery. Again, participants’ expectations were rated highly and regardless of the high expectation evident in the pre-survey all items did perform and scored higher in the post-survey. Of note was that participants rated the question on their perceived difficulty in adjusting to academic life lowest (39.2%) in the pre-survey and while it remained lowest in the post-survey it had increased to 78.4% indicating the difficulty participants have in balancing academic work, their daily work and family life. This is surprising given that 87.3% of the participants held a degree level or above. However, 87.3% were 11 years or more post qualification and participants’ difficulty may relate to their time out of education. The overall scoring of each item on the scale is presented in Table 4.

The third scale measured participants’ expectations regarding the programme upon completion and pre- and post-programme delivery. Again, participants’ expectations were rated highly and regardless of the high expectation evident in the pre-survey all items did perform and scored higher in the post-survey. Of note was that participants rated the question on the possibility of the programme increasing their responsibility lowest (53.9%) in the pre-survey and while it remained lowest in the post-survey it had increased to 87.3% indicating that because of the programme participants perceived their level of responsibility had increased. The overall scoring of each item on the scale is presented in Table 4.

Data from the qualitative open questions addressed; learning, anything participants would change, topics that participants would like included in the programme, the forum day, and general comments.

Qualitative comments regarding learning (n = 40) from the programme were positive and revolved around learning, the opportunity to meet other leaders, reflection, and linking theory and practice:

*Thought provoking. good to have time to think about my role, very enjoyable, great to meet with people from different services. learning to self-evaluate and link it to everyday practice, it was very good and interesting with a good balance given between information and reflection.*

Qualitative comments regarding anything they would change (n = 28) revolved around the assignment and group work and the one thing suggested for change was the aspect of including parents/family in decision-making and care provision:

*‘More clarification on what overall assessment is, I thought I would do my own assignment, and group work was difficult at first, however, a practical example was very helpful’.*

*‘Would like more discussion on parent/family involvement, how to support it, how to lead it and how to role model it for staff’.*

Qualitative comments regarding topics that participants would like to be included (*n* = 15) revolved around the priority of topics rather than topics to be addressed. Participants were generally happy with the content and comment suggested participants knew the topics would be covered but emphasised the ongoing need for support around leadership issues and that having time as a group to interact and discuss topics as a peer support system would be beneficial:

*We can’t get enough support on issues like ‘Supervision’ ‘Time management’ ‘Management conflict’ ‘Motivating the team’, ‘Team building’, ‘Engagement of frontline staff’, ‘Change Management’, ‘Promoting good leadership’, ‘Mentorship’ and ‘Reflective thinking’.*

*‘Allocate extra time to the groups as it’s difficult to get it all done and meet as a group as there are things you need to discuss as a group of peers that you need to bounce off others before you bring to the facilitators’.*

Qualitative comments regarding the forum day (n = 34) acknowledged it as a key source of learning and sharing where participants presented their group project. A broad range of projects were addressed, and key areas addressed by participants across groups were regulation aspects (inspection, preparedness), supervision (process, engagement, recording) person-centredness (choice, goal setting, transition, quality of life, decision making, quality improvements (communication, paperwork, handover, team meetings) and staff support (debriefing, stress). Participants highly valued the forum day as it presented an opportunity to share their work gain peer validation and gain from other participants’ projects and take ideas back to practice and highlight:

*‘This was a great day; I gained so much from it and all the presentations, I got so much from working with the group as we were all from different services and while it was difficult at the start to choose something, we could all do in our own service, in the end we did something of value to each of our services for the clients/service users and staff’.*

*‘I got some much from doing the group project but the real benefit was seeing and hearing the other groups as there was so much of their work that I can bring back to my area and want to introduce now also’.*

Qualitative comments addressing general comments related to having clear information before the programme (n = 5), factors influencing their decision to undertake the programme (n = 8), and the programme meeting their expectations (n = 22). Regarding having clear information participants expressed their desire and motivation for programmes to meet their needs and that the programme was undersold in their organisation. In terms of the decision to undertake the programme participants expressed their desire to ‘*learn from others’, ‘update knowledge’, ‘improve their leadership’*, and *‘to be effective and confident’*. Regarding expectations participants expressed their appreciation for the programme and were happy to have undertaken it and that it had sparked their interest and passion:

*‘Very beneficial to my current role, fantastic meeting others and networking a fantastic course and support I will miss it but have made valuable contacts which I will use in the future I have gained great insight and knowledge into areas as I have found the topics in the course thought-provoking’.*

*‘An excellent programme which has greatly increased my confidence and knowledge, great learning and shared opportunities, it has highlighted the importance of leadership and it has developed my way of thinking, the language I use and has brought me back to the beginning of being person-centred’.*

*‘I would be interested in doing another module like this as it has positively impacted on my career and learning as it increased my self-awareness and knowledge of how to communicate more effectively with colleagues’.*

## Discussion

Overall, the programme was positively evaluated with all aspects increasing from pre to post programme. However, while this increase is small it is nonetheless important, and given that 70% of the sample had 11^+^ years in service the pre-scores may have been more positively reported and indicate leadership programmes may have greater value for those in the early career stage (0–8 years). The co-design aspect of the programme ensured a focus on person-centredness, participants’ experience/s, and viewing participants as shared decision-makers [29]. Fundamental to the co-design of the programme delivery was drawing on the experience of participants and recognising the importance of reflective practice [30]. This was embedded throughout the programme where facilitators utilised methods that sought to draw on the participants’ aesthetic experience of what and how it feels like to use or be part of the service. Within the programme participants initially perceived leadership as mostly task-oriented but came to realise that in the reality of current practice and service provision qualities such as communication, person-centredness, advocacy, supporting, role modelling and empowering are key to modern leadership and that leadership is regarded as a distinct field and separate from management [31]. It’s important to consider certain limitations when interpreting the results. Firstly, the absence of a validated survey tool may have impacted the reliability and validity of the data collected. Secondly, the use of a cross-sectional design, rather than a longitudinal one, limits our ability to assess changes over time and draw causal conclusions. Thirdly, to truly ensure the programme philosophy is achieved a follow-up study would be recommended. Fourthly, the self-selection process may have attracted participants interested, eager, and motivated to engage in the programme and leadership. Additionally, the involvement of authors in delivering the programme and participants being aware that their responses, while anonymous, would be known to the research team, may have influenced participants’ responses, and resulted in a positively biased response.

Nonetheless, this programme emphasises the crucial need for support in transitioning service provision to a community-based model, which necessitates a cultural shift and the identification of leaders at an organisation level capable of leading change. Change within intellectual disability services has long been anticipated, yet progress remains slow, and much remains to be done [32]. The evolution of leadership presents a challenge, particularly in congregated settings where a top-down model of organisational management exists. In addition, a challenge for community-based settings staff will be working in small teams where managerial support is more remote [33]. Without organisational support and adequate education, there may be an apprehension and reluctance to assume leadership roles, this is seen anecdotally in the difficulty to recruit to leadership/PIC positions. However, the findings of this study suggest that with adequate support, time for reflection, and networking opportunities, participants can develop confidence and interpersonal leadership skills necessary for navigating change. Supportive measures such as mentoring, role modelling, and empowerment contribute significantly to this process.

Changes in policy, legislation, and service delivery pose both challenges and opportunities necessitating a re-evaluation of leadership and the need to enhance leadership capacity, adapt to contextual demands, and fulfil responsibilities [34]. This programme has made strides in addressing these challenges by fostering capacity, framing leadership within current issues, recognising leadership at various levels, and nurturing leadership qualities among staff. Moreover, participants grew to realise their role within the system and how they could influence developments through their encounters, both formal and informal, with staff and within their organisation [35]. Consequently, leadership is viewed as relational [34] aligning with the holism, person-centredness, advocacy-focused, and empowering models of care and support within intellectual disability services. However, for many participants in the programme, this realisation only occurred after providing them with the time and space away from work to reflect and develop.

In the dynamic landscape of service provision landscape characterised by advances in technology, economic fluctuations, and shifting policy direction, swift change can be unsettling for both staff and clients/service users. Therefore, investment in education and training to augment employees’ personal and professional development is crucial in managing change effectively and fostering a sense of belonging and engagement. Personal development and growth are important and participants in this programme articulated evidence of personal growth regarding increased confidence and motivation through engagement with the programme. Recognising that change can be stressful leading to skill reduction or feelings of confusion, being overwhelmed, and being undervalued [36–38]. Leaders play a vital role in influencing individuals and groups through their behaviours, perceptions, thoughts, and beliefs [39, 40]. Thereby, it is vital to cultivate leaders who can adapt their leadership style and principles to suit the goals, context, and characteristics of their team/s. Effective leaders are those who can learn, evolve, and navigate challenges with experience, thereby achieving results even in difficult situations [41]. 

Personal growth facilitated by self-awareness fosters a more accepting and compassionate self-view of oneself, enhances leadership capacities, and reshapes leaders’ values in navigating the change process [42]. Good leadership involves credibility through regular consistent feedback, role modelling, active presence in observing staff [43], and providing support [44]. Thus, leadership involves skilled professional guidance, instruction support, and an educational role that extends beyond mere direction [44]. Essential within this process is fostering dialogue between leaders, staff, and clients/service users, promoting mutual exploration of relationships [45]. This requires leaders, to articulate and communicate a clear vision, enhance knowledge and understanding through productive engagement, empowering others, providing feedback and reinforcement, and grounding new approaches [46]. Such an approach supports the distribution of responsibility, ensuring that rewards are collectively owned rather than individualistic. Effective change management hinges on strong communication strategies, early consultation with all stakeholders, and generating enthusiasm for change [47]. These strategies help mitigate against resistance and enhance implementation success [48] and encourage active participation and engagement of staff in the change process [49]. 

Within the programme, a key element in fostering effective communication was participants understanding of individual personality types and communication styles. The relationship between leaders and their teams’ hinges on trust, mutual respect, understanding, and the sharing of information. Strong leaders can empower individuals and teams to identify their learning needs, thus enhancing self-motivation and empowerment, crucial for sustaining a lifelong commitment to learning and instigating a cultural shift [50]. Strong organisational leadership plays a vital role in supporting educational opportunities for leaders tailored to the priorities of intellectual disability services. This enables leaders to motivate and maximise the benefit for individuals, staff, client/service users, and the overall service [51, 52]. Evidence suggests that strong leadership and role modelling involve promoting continuous professional development for individual staff [53], facilitating mentorship programmes [50], and empowering team members to contribute to service improvement initiatives [53, 54]. 

A three-pronged approach focusing on education, leadership, and practice cultivates an environment conducive to inquiry, facilitating and promoting evidence-based practice [52]. Investment in equipping leaders with the skills to provide high-quality, transformational, mentorship and leadership. Continuous professional development holds significant relevance as evidenced by the impact on participants. Such impact can influence one’s self-motivation, enhance practical relevance and application, affect workplace learning, foster enabling leadership, and contribute to a positive workplace culture [55]. However, while participants acknowledged the value of continuous professional development, their primary exposure to it, beyond this programme, often revolved around mandatary training provided through in-service programmes. The lack of outward continuous professional development initiatives may contribute to a sense of isolation, as articulated by participants who valued the opportunity to connect with peers from other services and the networking opportunities this fostered.

Effective leadership is fundamental to providing integrated health and social care [56], improving performance [57, 58], ensuring quality care [59], and fostering organisational commitment [60]. Research across health and social care has established a connection between leadership and client/service user care outcomes, including patient mortality rates [61, 62], medication errors [62], hospital-acquired infections [62], patient outcomes [19], and higher patient satisfaction [62]. Such correlations may be linked to supportive leadership fostering better work environments, improved resources, appropriate staffing levels, and effective care practices [19, 60, 63]. Given that change is unavoidable leaders must harness the knowledge, abilities, and skills of all team members, acknowledging that expertise can emerge from various sources within the team [64]. Such an approach necessitates a shift away from hierarchical managerial structures towards fostering and developing individuals as reflexive leaders [51, 65]. Leadership education should strike a balance between individual development and wider service priorities to ensure the efficient delivery of person-centred, safe, and effective care [55]. Crucial to effective leadership is cultivating a positive workplace culture [66], knowing what needs to change, and implementing practical strategies for enacting change [67, 68]. All members of staff hold some level of responsibility and it is advocated that leadership training be extended to all staff members [69]. This emphasis on leadership training is further reinforced by the focus on vulnerability and risk management embedded in policies and standards overseen by HIQA in Ireland.

Within the programme, participants were primarily focused on crisis-avoiding/managing and fulfilling the criteria set out by the funding and monitoring bodies. This position inhibits the potential for leadership to emerge and flourish, often resulting in ineffective adherence to policies and standards and a heightened risk of care erosion. Instances of such shortcomings have been observed in scandals in Ireland (70,71) and the UK [72] where the absence or failure of leadership has had profound consequences for all. Thereby, leadership is imperative at various levels within intellectual disability services and should be considered a role and responsibility of all staff. The diverse skills and strengths present within an organisation represent a vast reservoir of untapped leadership talent [34]. Recognising the contribution and value of all staff in a team necessitates a departure from the notion of concentrated leadership in the hands of a few, in favour of a more devolved leadership model and a collective decision-making approach. This entails expanding the pool of potential leaders and ensuring leadership emerges from collective efforts rather than relying solely on individual capabilities [73]. To accomplish this, there is a need to empower others to take initiative [74, 75] and prioritise the support and empowerment of leaders, so they can in turn support and empower others. This reframes leadership as a collaborative endeavour focused on partnership, engagement, and shared responsibility, transcending hierarchical power dynamics and seniority-based perspectives within the health and social care hierarchy [76]. 

Practice leadership in intellectual disability services has been studied and documented in both Australia and the UK [12, 13, 43, 77–79], with a focus on improving the quality of life for individuals with intellectual disability. A key concept that has emerged in this literature is the implementation of “active support,” which involves empowering individuals with disabilities to engage in meaningful activities and make choices in their daily lives, with appropriate support from staff members [77–79]. Practice leaders, including frontline managers and supervisors, play a pivotal role in creating a positive organisational culture that values person-centred care, fosters staff empowerment, and promotes continuous learning and improvement. Studies have shown that practice leaders who effectively implement active support report better staff experiences of working in challenging environments.

## Conclusion

While service models have evolved towards a more person-centred approach, intellectual disability services, and their leaders still face challenges in the transition to modern leadership paradigms. These new approaches emphasise individualised support, self-determination, effective teamwork, and evidence-based practice, which are integral to developing inclusive and participatory human service models focused on supporting individuals in achieving their goals. Effective leadership serves as a catalyst for organisational change and is closely associated with delivering safe, effective quality care and promoting positive outcomes. (80,81) Despite widespread research into management and leadership in health and social care services, the field of intellectual disability remains relatively under-researched, often described as both an important and neglected area [82]. 

Effective leadership improves outcomes for clients/service users and what is evident within this evaluation is that intellectual disability leaders are eager for change and improvement but may lack clarity on how to enact such changes. Moreover, providing high-quality personalised care presents challenges, thereby there is a need to invest in leader development and illustrate how their skills can drive service improvement, engage clients/service users in care planning, and embed person-centred care across the health and social care system. There is a pressing need to create and nurture practice leaders within intellectual disability services and relay the message that leadership is a shared responsibility among all individuals striving to enhance people’s care experiences, particularly for those who are most vulnerable. This involves transforming services into human-centred care models focused on fulfilling person-centred outcomes.^83.84^ Leaders play a pivotal role in promoting well-being, supporting independence, and ensuring people experience high-quality care and the best possible health outcomes. Overall, this evaluation reveals that participants’ knowledge and understanding of leadership improved through this programme and participants’ experiences were largely positive. However, consideration needs to be given to how leadership interventions related to support for individuals with intellectual disability, and future programme evaluation need to consider staff practice and service users’ experience has been measured.

**Strengths and limitations of this study**

- This study captures the development and evaluation of a leadership programme for staff working in intellectual disability services with person in charge responsibilities in Ireland.
- A large sample was achieved across four cohorts of the programme delivery and both survey and interview data were collected.
- The survey instrument was developed for this study but was not a valid tool.
- Social desirability bias from participants in their responses may have influenced their responses.

### Electronic supplementary material

Below is the link to the electronic supplementary material.

Supplementary Material 1

Supplementary Material 2

## Data Availability

Dataset is available on figshare open platform https://doi.org/10.6084/m9.figshare.22013186 and any additional information can be made to the corresponding author dependent upon privacy or ethical restrictions.

## Declarations

### Patient and public involvement

It was not appropriate or possible to involve patients or the public in the design, conduct, reporting, or dissemination plans of our research as this was a commissioned evaluation. However, stakeholders were involved throughout the design and development of the programme.
