# Supplementary material for: Leadership in intellectual disability practice: design, development, and evaluation of a programme to support practice
Source: BMC Health Serv Res. 2024 May 28;24:674. doi: 10.1186/s12913-024-11124-7 (PMC11134711; doi:10.1186/s12913-024-11124-7)
Supplement: Supplementary file 2 — Supplementary Material 2 [file 12913_2024_11124_MOESM2_ESM.doc]

**Questionnaire Pre**

**Section One – Demographic Details**

***For all questions, please tick the appropriate box***

1. **Age category**

20- 30 years  31 - 40 years  41 – 50 years  51 – 60 years  60+ years

1. **Gender**

Male  Female

1. **Work pattern**

Full-Time  Part-Time  Other (Please specify) ___________________________

1. **Which of the following best describes your current work pattern?**

Day Duty (no weekends)  Day Duty (including weekends)  Nights Duty

Shift work (no nights)  Shift work (including nights)

1. **Within which service/practice area are you currently based?**

Please specify ____________________________**______________**

1. **Please indicate your current role**

Registered Nurse  Nurse Manager  Clinical Nurse Specialist  Social Care Worker  Social Care Manager

1. **Professional Qualifications (Please tick all that apply)**

Certificate  Degree  Post Graduate  Masters

Clarify ___________________________________________________________________________________

**Q8 Number of years nursing experience?**

1 to 5 years  6 to 10 years  11 to years 20  21+ years

**Q9. Where did you first hear about the programme?**

______________________________________________________________________________________________________________________________________________________________________________________________________________________________________________________________________________

**Q10. Where did you gain information on the programme?**

______________________________________________________________________________________________________________________________________________________________________________________________________________________________________________________________________________

____________________________________________________________________________________________________________________________________________________________________________________

**Q11. What factors influenced you in deciding to enroll on the programme?**

______________________________________________________________________________________________________________________________________________________________________________________________________________________________________________________________________________

____________________________________________________________________________________________________________________________________________________________________________________

______________________________________________________________________________________________________________________________________________________________________________________________________________________________________________________________________________

**Q12. Did you receive support from Management in your organisation?**

Yes  No  (please elaborate)

________________________________________________________________________________________________________________________________________________________________________________________________________________________________________________________________________________________________________________________________________________________________________

**Q13. What were you expectations of the programme?**

______________________________________________________________________________________________________________________________________________________________________________________________________________________________________________________________________________

____________________________________________________________________________________________________________________________________________________________________________________

______________________________________________________________________________________________________________________________________________________________________________________________________________________________________________________________________________

**Section Two – Prior to commencing the programme**

*For all questions, please tick the appropriate box a rating scale applies as follows*:

strongly agree; agree; neither agree nor disagree; disagree; strongly disagree

| **Question** | | **Strongly**  **Agree** | **Agree** | **Neither**  **Agree nor Disagree** | **Disagree** | **Strongly Disagree** |
| --- | --- | --- | --- | --- | --- | --- |
| **Q.14** | **Information regarding the programmes was easily accessible** | 🞎 | 🞎 | 🞎 | 🞎 | 🞎 |
| **Q.15** | **I had sufficient information regarding the course to make my decision to enroll** | 🞎 | 🞎 | 🞎 | 🞎 | 🞎 |
| **Q.16** | **I am confident that the programme will assist me in my practice** | 🞎 | 🞎 | 🞎 | 🞎 | 🞎 |
| **Q.17** | **I am confident that the programme will assist me in my future career** | 🞎 | 🞎 | 🞎 | 🞎 | 🞎 |
| **Q.18** | **I am undertaking the programme to improve my knowledge and skill** | 🞎 | 🞎 | 🞎 | 🞎 | 🞎 |
| **Q.19** | **I am undertaking the programme to assist my prospect of promotion** | 🞎 | 🞎 | 🞎 | 🞎 | 🞎 |
| **Q.20** | **I am undertaking the programme as part of my own personal development** | 🞎 | 🞎 | 🞎 | 🞎 | 🞎 |
| **Q.21** | **I am undertaking the programme as it would give me a sense of greater job satisfaction** | 🞎 | 🞎 | 🞎 | 🞎 | 🞎 |
| **Q.22** | **I am undertaking the programme to benefit the organisation in which I work** | 🞎 | 🞎 | 🞎 | 🞎 | 🞎 |

**Section Three – Expectation during the programme**

*For all questions, please tick the appropriate box a rating scale applies as follows*:

strongly agree; agree; neither agree nor disagree; disagree; strongly disagree

| **Question** | | **Strongly**  **Agree** | **Agree** | **Neither**  **Agree nor Disagree** | **Disagree** | **Strongly Disagree** |
| --- | --- | --- | --- | --- | --- | --- |
| **Q.23** | **The content of the modules will be appropriate to my practice** | 🞎 | 🞎 | 🞎 | 🞎 | 🞎 |
| **Q.24** | **An introduction will be provided to all relevant resources** | 🞎 | 🞎 | 🞎 | 🞎 | 🞎 |
| **Q.25** | **Relevant resources will be available to support me undertake the programme** | 🞎 | 🞎 | 🞎 | 🞎 | 🞎 |
| **Q.26** | **I will find it difficult to adjust to academic life** | 🞎 | 🞎 | 🞎 | 🞎 | 🞎 |
| **Q.27** | **I will apply my learning to practice** | 🞎 | 🞎 | 🞎 | 🞎 | 🞎 |
| **Q.28** | **Moodle will be a valuable resource for learning** | 🞎 | 🞎 | 🞎 | 🞎 | 🞎 |
| **Q.29** | **Facilitators on the programme will be supportive** | 🞎 | 🞎 | 🞎 | 🞎 | 🞎 |
| **Q.30** | **I will ask questions related to topics been discussed** | 🞎 | 🞎 | 🞎 | 🞎 | 🞎 |
| **Q.31** | **I will manage my time well** | 🞎 | 🞎 | 🞎 | 🞎 | 🞎 |
| **Q.32** | **Assessments will be appropriate** | 🞎 | 🞎 | 🞎 | 🞎 | 🞎 |
| **Q.33** | **I am aware of the time necessary for the programme** | 🞎 | 🞎 | 🞎 | 🞎 | 🞎 |

**Section Four - Expectation upon completion of the programme**

*For all questions, please tick the appropriate box a rating scale applies as follows*:

strongly agree; agree; neither agree nor disagree; disagree; strongly disagree

| **Question** | | **Strongly**  **Agree** | **Agree** | **Neither**  **Agree nor Disagree** | **Disagree** | **Strongly Disagree** |
| --- | --- | --- | --- | --- | --- | --- |
| **Q.34** | **I will gain knowledge relevant to person in charge** | 🞎 | 🞎 | 🞎 | 🞎 | 🞎 |
| **Q.35** | **My analytical skills will develop** | 🞎 | 🞎 | 🞎 | 🞎 | 🞎 |
| **Q.36** | **I will gain a more comprehensive view of client care** | 🞎 | 🞎 | 🞎 | 🞎 | 🞎 |
| **Q.37** | **I will become more aware of the evidence behind practice** | 🞎 | 🞎 | 🞎 | 🞎 | 🞎 |
| **Q.38** | **I will develop my skills in evaluating evidence** | 🞎 | 🞎 | 🞎 | 🞎 | 🞎 |
| **Q.39** | **I will become skilled in evaluating my own practice** | 🞎 | 🞎 | 🞎 | 🞎 | 🞎 |
| **Q.40** | **I will be able to implement change in my practice area** | 🞎 | 🞎 | 🞎 | 🞎 | 🞎 |
| **Q.41** | **My education will positively impact on client care** | 🞎 | 🞎 | 🞎 | 🞎 | 🞎 |
| **Q.42** | **I will become more confident talking to clients/client families about care** | 🞎 | 🞎 | 🞎 | 🞎 | 🞎 |
| **Q.43** | **I will apply my learning in my workplace** | 🞎 | 🞎 | 🞎 | 🞎 | 🞎 |
| **Q.44** | **I will gain confidence in communicating with my colleagues/healthcare members** | 🞎 | 🞎 | 🞎 | 🞎 | 🞎 |
| **Q.45** | **I will gain confidence in speaking at team meetings** | 🞎 | 🞎 | 🞎 | 🞎 | 🞎 |
| **Q.46** | **I will share my knowledge with other colleagues** | 🞎 | 🞎 | 🞎 | 🞎 | 🞎 |
| **Q.47** | **I will become more innovative in my practice** | 🞎 | 🞎 | 🞎 | 🞎 | 🞎 |
| **Q.48** | **I will become more confident advocating for clients** | 🞎 | 🞎 | 🞎 | 🞎 | 🞎 |
| **Q.49** | **The programme will positively impact my work** | 🞎 | 🞎 | 🞎 | 🞎 | 🞎 |
| **Q.50** | **The programme will increase my satisfaction with my work** | 🞎 | 🞎 | 🞎 | 🞎 | 🞎 |
| **Q.51** | **The programme will will have a positive impact on my career** | 🞎 | 🞎 | 🞎 | 🞎 | 🞎 |
| **Q.52** | **The programme will increase my responsibility** | 🞎 | 🞎 | 🞎 | 🞎 | 🞎 |

**Section Five - Additional Comments**

| **Q.53** | **Anything you would like to add. _____________________________________________________________________________________________________________________________________________________________________________________________________________________________________________________________________________________________________________________________________________________________________________________________________________________________________________________________________________________________________________________________________________________________________________________________________________________________________________________________________________________________________________________________________________________________________________________________________________________________________________________________________________________________________________________________________________________________________________________________________________________________________________________________________________________________________________________________________________________________________________________________________________________________________________________________________________________________________________________________________________________________________________________________________________________________________________________________________________** |
| --- | --- |

***Thank you for taking time to complete this questionnaire, please place it in the stamped addressed envelop enclosed and return to the researchers.***

**Questionnaire Post**

**Section One – the Programme**

*For all questions, please tick the appropriate box a rating scale applies as follows*:

strongly agree; agree; neither agree nor disagree; disagree; strongly disagree

| **Question** | | **Strongly**  **Agree** | **Agree** | **Neither**  **Agree nor Disagree** | **Disagree** | **Strongly Disagree** |
| --- | --- | --- | --- | --- | --- | --- |
| **Q.1** | **Information regarding the programmes was easily accessible** | 🞎 | 🞎 | 🞎 | 🞎 | 🞎 |
| **Q.2** | **I had sufficient information regarding the course to make my decision to enroll** | 🞎 | 🞎 | 🞎 | 🞎 | 🞎 |
| **Q.3** | **I am confident that the programme has assisted me in my practice** | 🞎 | 🞎 | 🞎 | 🞎 | 🞎 |
| **Q.4** | **I am confident that the programme willassist me in my future career** | 🞎 | 🞎 | 🞎 | 🞎 | 🞎 |
| **Q.5** | **The programme has improved my knowledge and skill** | 🞎 | 🞎 | 🞎 | 🞎 | 🞎 |
| **Q.6** | **The programme will assist my prospect of promotion** | 🞎 | 🞎 | 🞎 | 🞎 | 🞎 |
| **Q.7** | **I have undertaken the programme as part of my own personal development** | 🞎 | 🞎 | 🞎 | 🞎 | 🞎 |
| **Q.8** | **The programme has given me a sense of greater job satisfaction** | 🞎 | 🞎 | 🞎 | 🞎 | 🞎 |
| **Q.9** | **The programme has benefited the organisation in which I work** | 🞎 | 🞎 | 🞎 | 🞎 | 🞎 |

**Section Two – Expectation upon completion of the programme**

*For all questions, please tick the appropriate box a rating scale applies as follows*:

strongly agree; agree; neither agree nor disagree; disagree; strongly disagree

| **Question** | | **Strongly**  **Agree** | **Agree** | **Neither**  **Agree nor Disagree** | **Disagree** | **Strongly Disagree** |
| --- | --- | --- | --- | --- | --- | --- |
| **Q.10** | **The content of the modules were appropriate to my practice** | 🞎 | 🞎 | 🞎 | 🞎 | 🞎 |
| **Q.11** | **An introduction was provided to all relevant resources** | 🞎 | 🞎 | 🞎 | 🞎 | 🞎 |
| **Q.12** | **Relevant resources were available to support me undertake the programme** | 🞎 | 🞎 | 🞎 | 🞎 | 🞎 |
| **Q.13** | **I found it difficult to adjust to academic life** | 🞎 | 🞎 | 🞎 | 🞎 | 🞎 |
| **Q.14** | **I am applying my learning to practice** | 🞎 | 🞎 | 🞎 | 🞎 | 🞎 |
| **Q.15** | **Moodle has been a valuable resource for learning** | 🞎 | 🞎 | 🞎 | 🞎 | 🞎 |
| **Q.16** | **Faciliators on the programme were supportive** | 🞎 | 🞎 | 🞎 | 🞎 | 🞎 |
| **Q.17** | **I was facilitated to ask questions related to topics being discussed** | 🞎 | 🞎 | 🞎 | 🞎 | 🞎 |
| **Q.18** | **I have managed my time well** | 🞎 | 🞎 | 🞎 | 🞎 | 🞎 |
| **Q.19** | **Assessments were appropriate** | 🞎 | 🞎 | 🞎 | 🞎 | 🞎 |
| **Q.20** | **The time necessary for the programme was realistic** | 🞎 | 🞎 | 🞎 | 🞎 | 🞎 |

**Section Three - Expectation upon completion of the programme**

*For all questions, please tick the appropriate box a rating scale applies as follows*:

strongly agree; agree; neither agree nor disagree; disagree; strongly disagree

| **Question** | | **Strongly**  **Agree** | **Agree** | **Neither**  **Agree nor Disagree** | **Disagree** | **Strongly Disagree** |
| --- | --- | --- | --- | --- | --- | --- |
| **Q.21** | **I have gained knowledge relevant to person in charge** | 🞎 | 🞎 | 🞎 | 🞎 | 🞎 |
| **Q.22** | **My analytical skills have been developed** | 🞎 | 🞎 | 🞎 | 🞎 | 🞎 |
| **Q.23** | **I have gained a more comprehensive view of client care** | 🞎 | 🞎 | 🞎 | 🞎 | 🞎 |
| **Q.24** | **I have become more aware of the evidence behind practice** | 🞎 | 🞎 | 🞎 | 🞎 | 🞎 |
| **Q.25** | **I have developed my skills in evaluating evidence** | 🞎 | 🞎 | 🞎 | 🞎 | 🞎 |
| **Q.26** | **I have become skilled in evaluating my own practice** | 🞎 | 🞎 | 🞎 | 🞎 | 🞎 |
| **Q.27** | **I will be able to implement change in my practice area** | 🞎 | 🞎 | 🞎 | 🞎 | 🞎 |
| **Q.28** | **My education will positively impact on client care** | 🞎 | 🞎 | 🞎 | 🞎 | 🞎 |
| **Q.29** | **I have become more confident talking to clients/client families about care** | 🞎 | 🞎 | 🞎 | 🞎 | 🞎 |
| **Q.30** | **I have applied my learning in my workplace** | 🞎 | 🞎 | 🞎 | 🞎 | 🞎 |
| **Q.31** | **I have gained confidence in communicating with my colleagues/healthcare members** | 🞎 | 🞎 | 🞎 | 🞎 | 🞎 |
| **Q.32** | **I have gained confidence in speaking at team meetings** | 🞎 | 🞎 | 🞎 | 🞎 | 🞎 |
| **Q.33** | **I have shared my knowledge with other colleagues** | 🞎 | 🞎 | 🞎 | 🞎 | 🞎 |
| **Q.34** | **I have become more innovative in my practice** | 🞎 | 🞎 | 🞎 | 🞎 | 🞎 |
| **Q.35** | **I have become more confident advocating for clients** | 🞎 | 🞎 | 🞎 | 🞎 | 🞎 |
| **Q.36** | **The programme has positively impacted my work** | 🞎 | 🞎 | 🞎 | 🞎 | 🞎 |
| **Q.37** | **The programme has increased my satisfaction with my work** | 🞎 | 🞎 | 🞎 | 🞎 | 🞎 |
| **Q.38** | **The programme has had a positive impact on my career** | 🞎 | 🞎 | 🞎 | 🞎 | 🞎 |
| **Q.39** | **The programme has increased my responsibility** | 🞎 | 🞎 | 🞎 | 🞎 | 🞎 |

**Section Four - Additional Comments**

|  | **Q40. Did the programme meet your expectations?**  __________________________________________________________________________________________________________________________________________________________________________________________________________________________________________________________________________________________________________________________________________________________________________________________________________________________________________________________________________________________________________________________________________________________________________________________________________________________________________________________________________________________________________________________________________________________________________________________________________________________________________________________________________________  ***Anything you would like to add. __________________________________________________________________________________________________________________________________________________________________________________________________________________________________________________________________________________________________________________________________________________________________________________________________________________________________________________________________________________________________________________________________________________________________________________________________________________________________________________________________________________________________________________________________________________________________________________________________________________________________________________________________________________________________________________________** |
| --- | --- |

***Thank you for taking time to complete this questionnaire.***

***Thanking you***
